# Reversible RNA Acylation Using Bio-Orthogonal Chemistry Enables Temporal Control of CRISPR-Cas9 Nuclease Activity

**DOI:** 10.1021/acschembio.4c00117

**Published:** 2024-07-25

**Authors:** Bhoomika Pandit, Linglan Fang, Eric T. Kool, Maksim Royzen

**Affiliations:** † Department of Chemistry, University at Albany, 1400 Washington Ave, Albany, New York 12222, United States; ‡ Department of Chemistry, Stanford University, 450 Serra Mall, Stanford, California 94305, United States

## Abstract

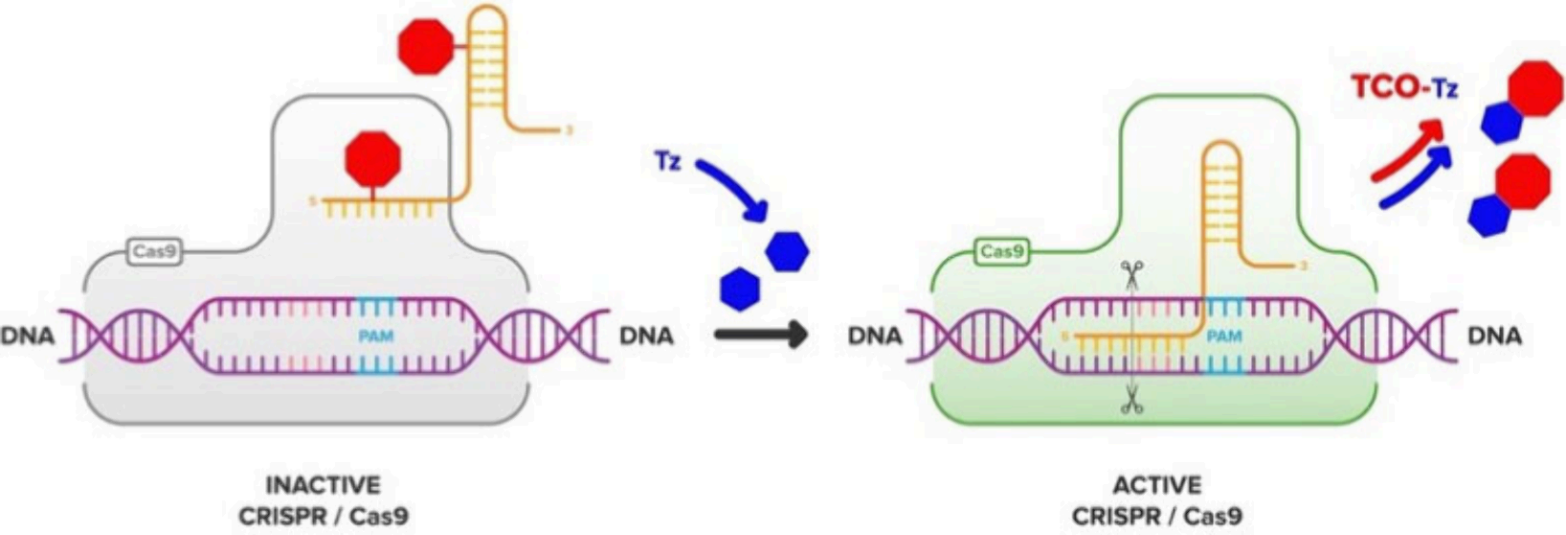

The CRISPR–Cas9 system is a widely popular tool for genome engineering. There is strong interest in developing tools for temporal control of CRISPR-Cas9 activity to address some of the challenges and to broaden the scope of potential applications. In this work, we describe a bio-orthogonal chemistry-based approach to control nuclease activity with temporal precision. We report a *trans*-cyclooctene (TCO)–acylimidazole reagent that acylates 2′–OH groups of RNA. Poly acylation (“cloaking”) of RNA was optimized *in vitro* using a model 18-nt oligonucleotide, as well as CRISPR single guide RNA (sgRNA). Two hours of treatment completely inactivated sgRNA for Cas9-assisted DNA cleavage. Nuclease activity was restored upon addition of tetrazine, which removes the TCO moieties via a two-step process (“uncloaking”). The approach was applied to target the GFP gene in live HEK293 cells. GFP expression was analyzed by flow cytometry. In the future, we anticipate that our approach will be useful in the field of developmental biology, by enabling investigation of genes of interest at different stages of an organism’s development.

## Introduction

The CRISPR (clustered regularly interspaced short palindromic repeats)–Cas9 system has become a widely employed tool for genome engineering in a broad range of organisms and systems because of its programmability, commercial availability, and ease of use.^1,2^ The most frequently used genome editing tool is the type II Cas9 from *Streptococcus pyogenes* strain SF370 (SpyCas9) and single guide RNA (sgRNA).^3,4^ Cas9 programmed with sgRNAs targets specific DNA sequences in the genome to create a blunt-ended double-strand break.^5^ In addition to engineering CRISPR-Cas9 for different biomedical applications, there has also been interest in developing strategies to regulate the CRISPR-Cas9 activity. Technologies that can inactivate CRISPR-Cas9 inside the cell can be used to minimize the off-target effects that occur on a slower time scale.^6,7^ Meanwhile, technologies that turn on a caged CRISPR-Cas9 system will find many important applications in developmental biology. For example, caged CRISPR-Cas9 systems can, in principle, be delivered to an organism, such as zebrafish, during early stages of embryogenesis. The CRISPR-Cas9 system will stay dormant until it is activated by an exogenous trigger at a particular stage of zebrafish development. This toolkit will allow to investigate a gene of interest during a particular stage of an animal’s development.^8^

Several approaches for temporal control of CRISPR-Cas9 activity have been reported. Temporal control of Cas9 function was shown by the Choudhary group. Cas9 was fused to the destabilized domains of *E. coli’s* dihydrofolate reductase.^9^ These domains are unstable and rapidly target the fusion protein for proteasomal degradation. The destabilized domains were stabilized upon the addition of the small molecule trimethoprim, which prevented proteasomal degradation. In addition to the described, alternative methods to modulate Cas9 activity have been reported by Bondy-Denomy and Yee groups.^10−12^ Temporal control of sgRNA has been shown by Batey and co-workers, who fused a theophylline-binding aptamer into the sgRNA’s tetraloop.^13^ The authors carried out a selection process that yielded constructs that exhibited nuclease activity only in the presence of theophylline. Kundert et al. reported a similar system containing sgRNA-fused aptamer which can be controlled by small molecule ligands.^14^ All of the described systems are synthetically complex and require considerable bioengineering.

## Results and Discussion

Herein we describe a different approach to control CRISPR-Cas9 activity using bond-breaking bio-orthogonal chemistry between *trans*-cylooctene (TCO) and 1,2,4,5-tetrazine-3,6-dimethanamine (**Tz**).^15^ As previously described, this chemistry consists of two steps: an inverse electron demand Diels–Alder cycloaddition, followed by an intramolecular cyclization.^15^ The approach is also based on the “RNA cloaking” strategy recently developed by Kool and co-workers.^16,17^ The strategy utilizes acylimidazole reagents to reversibly modify the 2′–OH groups of functional RNAs, which results in blocking of RNAs’ folding, hybridization, and protein interactions. A number of different cloaking reagents have been reported in recent years.^17,18^ Subsequent uncloaking has been accomplished using light, nucleophiles, or phosphine reagents. The reversible acylation strategy has been shown to enhance the RNA stability by protecting it from chemical and enzymatic degradation. Reversible acylation has also been utilized to trap transient RNA complexes.^19^ Previously, acylation approaches to caging CRISPR sgRNAs involved the use of an azide-containing reagent, which required the use of phosphines to remove acyl groups to restore RNA function;^20,21^ this strategy is limited by the relatively slow rate of uncaging and by the toxicity of the phosphines.

**Figure 1:**
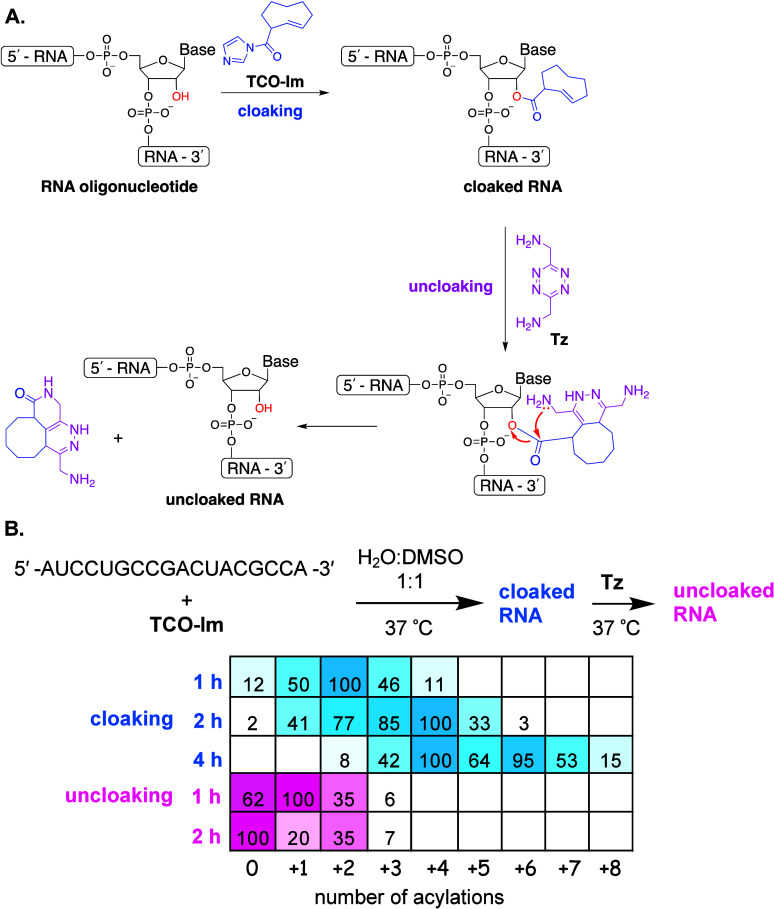
(**A**.) The chemical mechanism of cloaking with **TCO-Im** and uncloaking using **Tz**; (**B**.) summary of the MALDI-TOF data.

To cloak RNA with a releasable TCO, we developed a TCO-acylimidazole reagent (**TCO-Im**), shown in Figure 1A. When reacting with RNA, it will install the TCO moiety onto multiple 2′–OH groups stochastically. Subsequent uncloaking can be accomplished using bond-breaking bio-orthogonal reaction with **Tz**, which contains nucleophilic amines, also illustrated in Figure 1A.^15^ The advantage of using the inverse electron demand Diels–Alder reaction between TCO and tetrazine (Tz) is that the two reagents have been shown to be nontoxic and compatible with many biological applications.^22−24^ We determined the second-order rate constant in the click reaction between **Tz** and methyl (2*E*)-2-cyclooctene-1-carboxylate to be 1.06 ± 0.04 M^–1^ sec^–1^ (Figure S1).

As a proof-of-concept, we initially treated a model 18-nt RNA oligonucleotide with **TCO-Im** (Figure 1B) for 1, 2, and 4 h at 37 °C. Because of poor solubility of **TCO-Im** in aqueous media, the reaction was done in a 1:1 solution of DMSO and H_2_O. Afterward, the RNA was purified by ethanol precipitation, and its acylation profile was analyzed by matrix-assisted laser desorption/ionization–time-of-flight (MALDI-TOF). The acquired MALDI-TOF spectra are shown in Figures S2–S4. As summarized in Figure 1B, nonspecific 2′-acylation of the model RNA with **TCO-Im** is fast. One-hour treatment produced a mixture of acylated RNAs each with a median number of two TCO groups attached. Longer exposure to the acylating reagent increases the extent of the RNA modification. Four-hour treatment produced a complex mixture of acylated RNAs with a median number of five TCO groups attached.

We subsequently tested the uncloaking of the model RNA that previously underwent cloaking for 4 h. The RNA was treated with **Tz** at 37 °C in PBS (pH 7.2). One hour of uncloaking was found to be insufficient (Figures 1B and S5). The proposed uncloaking mechanism consists of two steps: an intramolecular click reaction between TCO and Tz, followed by an intramolecular cyclization (Figure 1A). At micromolar concentrations, the release step has been shown to be slower than the click step.^25,26^ MALDI-TOF analysis of the 1 h with the **Tz** reaction showed mono- and di-**Tz** adducts that did not undergo the intramolecular cyclization step. Two hours of uncloaking was found to increase the yield of fully uncloaked RNA (Figures 1B and S6).

**Figure 2:**
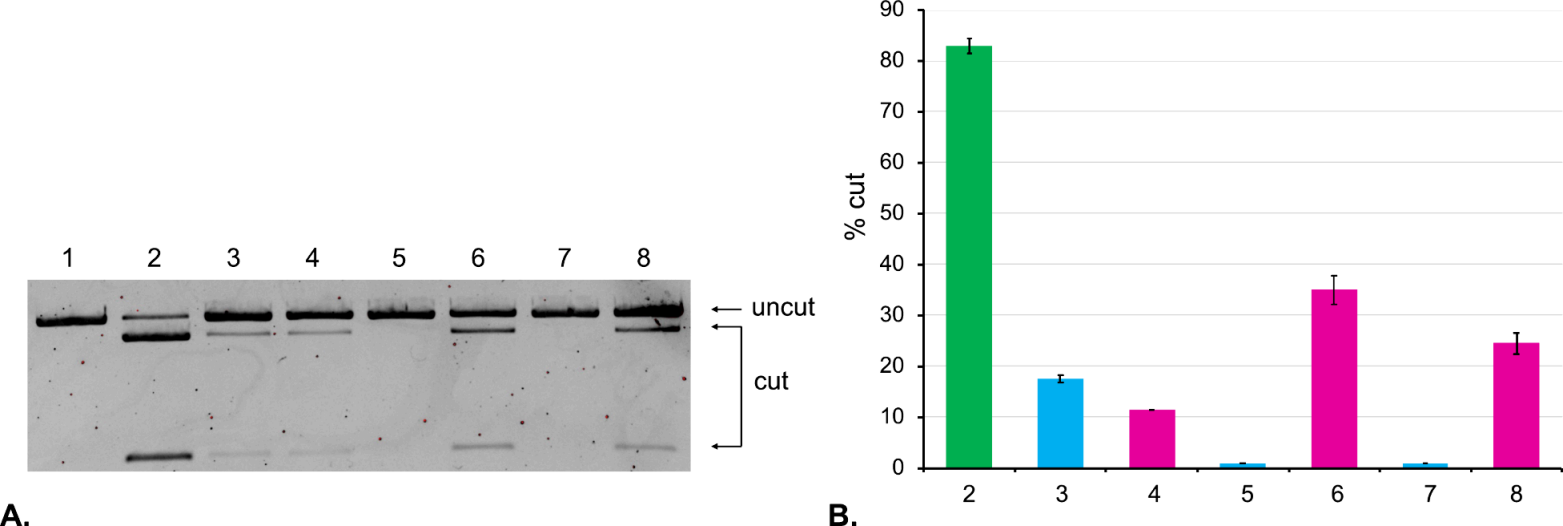
Analysis of CRISPR-Cas9 experiments using agarose gel electrophoresis. (**A**) *Lane 1*: linearized eGFP-N1 plasmid; *Lane 2*: linearized eGFP-N1 plasmid and **sgRNA 1**; *Lane 3*: linearized eGFP-N1 plasmid and **sgRNA 1** cloaked with **TCO-Im** for 1 h; *Lane 4*: linearized eGFP-N1 plasmid and **sgRNA 1** cloaked with **TCO-Im** for 1 h and uncloaked with **Tz** for 1 h; *Lane 5*: linearized eGFP-N1 plasmid and **sgRNA 1** cloaked with **TCO-Im** for 2 h; *Lane 6*: linearized eGFP-N1 plasmid and **sgRNA 1** cloaked with **TCO-Im** for 2 h and uncloaked with **Tz** for 2 h; *Lane 7*: linearized eGFP-N1 plasmid and **sgRNA 1** cloaked with **TCO-Im** for 4 h; *Lane 8*: linearized eGFP-N1 plasmid and **sgRNA 1** cloaked with **TCO-Im** for 4 h and uncloaked with **Tz** for 4 h. (**B**) Bar chart representing the percentage of cut DNA upon treatment with the constructs in part **A**. Lane numbering is the same as in part **A**. All CRISPR experiments were performed in duplicate. Error bars represent ± s.d.

We assessed if our approach allows control of CRISPR-Cas9 activity using bio-orthogonal chemistry. We carried out acylation of **sgRNA 1** that was designed to target linearized eGFP-N1 plasmid. **sgRNA 1** was treated with **TCO-Im** for 1, 2, and 4 h at 37 °C and was subsequently purified by ethanol precipitation. To investigate the impact of cloaking, we carried out CRISPR experiments by treating the linearized eGFP-N1 plasmid with the acylated sgRNAs and Cas9 for 16 h. The ability of cloaked RNAs to carry out Cas9-assisted cleavage of dsDNA was analyzed by agarose gel electrophoresis, shown in Figure 2A. Lane 2 shows activity of the native **sgRNA 1**. One hour of cloaking with **TCO-Im** lowered the CRISPR-Cas9 activity more than 4-fold (lane 3). Cloaking for 2 and 4 h completely eliminated any observable nuclease activity (lanes 5 and 7). Uncloaking experiments were carried out by treating each of the cloaked **sgRNA 1** with **Tz** at 37 °C in PBS (pH 7.2). CRISPR-Cas9 activity of the uncloaked sgRNAs was once again analyzed by agarose gel electrophoresis, shown in Figure 2A (lanes 4, 6, and 8). We were unable to fully restore the native CRISPR-Cas9 activity, likely because **sgRNA 1** is considerably longer than the 18-nt model RNA. It is expected to have a more complex acylation profile that will take a longer time to fully uncloak. All CRISPR experiments were performed in duplicate. The data shown in Figure 2A was quantitated using ImageJ software and plotted in Figure 2B. Based on our experimental data, cloaking for 2 h, followed by uncloaking for 2 h showed the best results for controlling CRISPR-Cas9 activity.

Our next quest was to apply our approach to control CRISPR-Cas9 activity in live GFP-expressing HEK293 cells. However, we were concerned about the post-transfection stability of sgRNA that might be exposed to nuclease degradation inside the cells. We addressed this concern by site-specific modification of sgRNA with 2′-OMe groups. We followed the strategy described by Yin et al., who identified the exact positions which can be modified with 2′-OMe without significant perturbation to native binding between sgRNA and Cas9.^27^ We synthesized **sgRNA 2**, having the following sequence:

5′-GGGCGAGGAGCUGUUCACCGGUUUUAGagcuagaaauagcaaGUUaAaAuAaggcuaGUccG

UUAucAAcuugaaaaagugGcaccgagucggugcuuuuu-3′

Capital letters indicate unmodified nucleotides, while small letters correspond to nucleotides containing 2′-OMe groups.

**Figure 3:**
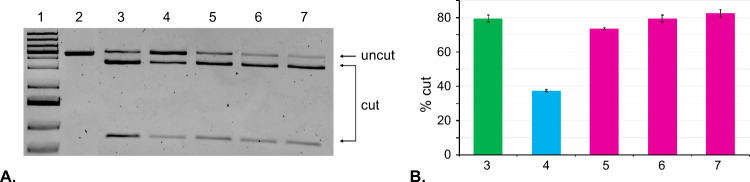
Analysis of CRISPR-Cas9 experiments using agarose gel electrophoresis. (**A**) *Lane 1*: DNA ladder; *Lane 2*: linearized eGFP-N1 plasmid; *Lane 3*: linearized eGFP-N1 plasmid and **sgRNA 2**; *Lane 4*: linearized eGFP-N1 plasmid and **sgRNA 2** cloaked with **TCO-Im** for 2 h; *Lane 5*: linearized eGFP-N1 plasmid and **sgRNA 2** cloaked with **TCO-Im** for 2 h and uncloaked with 50 mM **Tz** for 2 h; *Lane 6*: linearized eGFP-N1 plasmid and **sgRNA 2** cloaked with **TCO-Im** for 2 h and uncloaked with 5 mM **Tz** for 2 h; *Lane 7*: linearized eGFP-N1 plasmid and **sgRNA 2** cloaked with **TCO-Im** for 2 h and uncloaked with 1 mM **Tz** for 2 h. (**B**) Bar chart representing the percentage of cut DNA upon treatment with the constructs in part **A**. Lane numbering is the same as in part **A**. All CRISPR experiments were performed in duplicate. Error bars represent ± s.d.

In preparation for cellular studies, we tested our best conditions for cloaking **sgRNA 2** with **TCO-Im** and subsequent uncloaking with **Tz**. The impact of cloaking was investigated by CRISPR experiments, treating the linearized eGFP-N1 plasmid with the acylated **sgRNA 2** and Cas9 for 16 h. The ability of acylated **sgRNA 2** to carry out Cas9-assisted cleavage of dsDNA was analyzed by agarose gel electrophoresis, shown in Figure 3A. Lane 3 corresponds to the CRISPR experiment with **sgRNA 2**. **sgRNA 2** acylated with **TCO-Im** for 2 h (lane 4) lost more than half of its CRISPR activity. The degree of inactivation is smaller than that observed with **sgRNA 1**. We believe that this is due to the fact that fewer 2′–OH groups are available for acylation in **sgRNA 2**, which contains 59 2′-OMe groups. Some of these “blocked” sites were acylated with **TCO-Im** in **sgRNA 1**, which caused interference with proper binding to Cas9. The incorporated 2′-OMe groups are smaller than TCO and do not impact the sgRNA-Cas9 complex to the same extent. We subsequently carried out uncloaking using three different concentrations of **Tz** (Figure 3A, lanes 5–7). After 2 h, all of the compounds restored the CRISPR activity of the system. All CRISPR experiments were performed in duplicate. The data shown in Figure 3A was quantitated using ImageJ software and plotted in Figure 3B.

**Figure 4:**
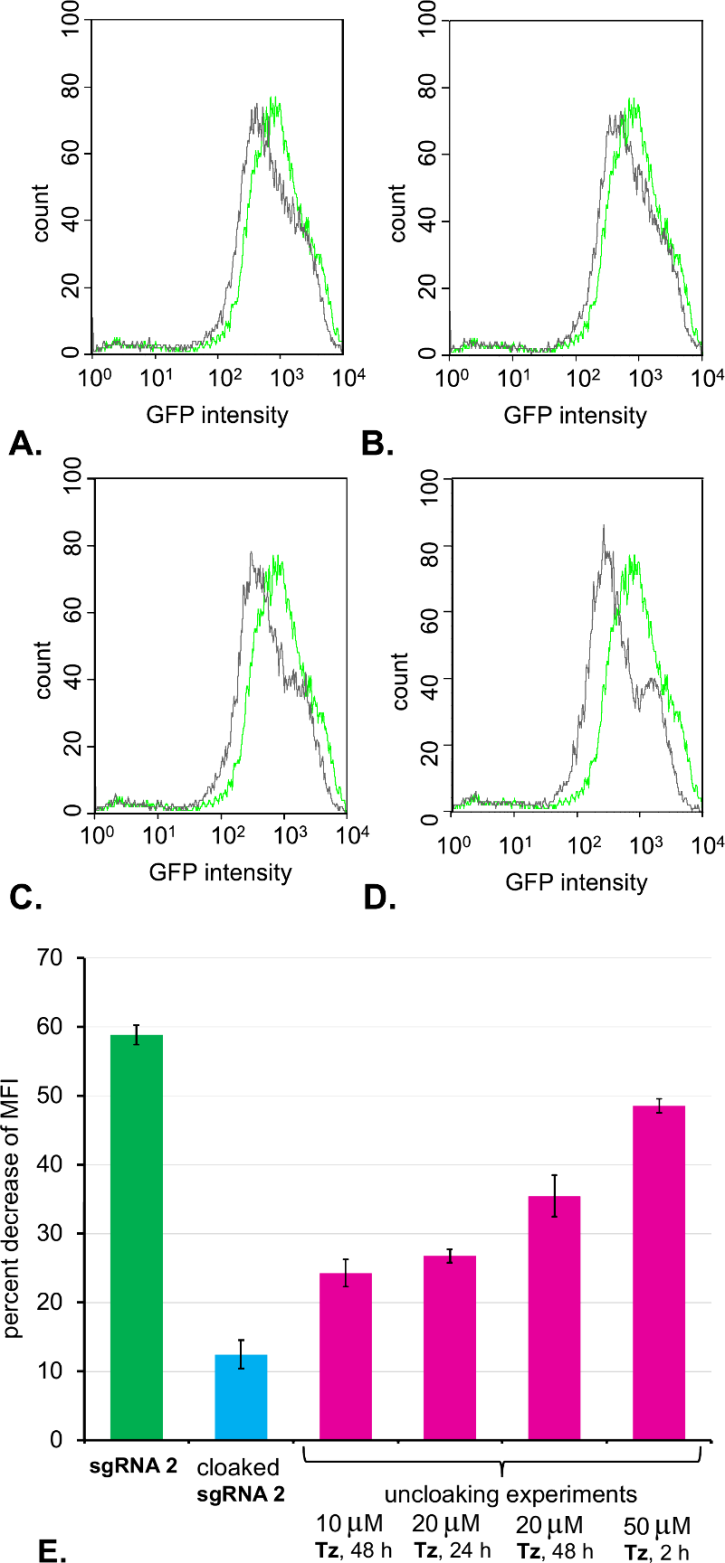
Flow cytometry analysis of CRISPR experiments targeting the GFP gene in HEK293 cells. Histograms of untreated GFP-expressing HEK293 cells are shown in green. Histograms of GFP-expressing HEK293 cells transfected with cloaked **sgRNA 2** followed by uncloaking with **Tz** are shown in gray. Uncloaking of **sgRNA 2** was optimized by varying the concentration of **Tz** and treatment times: (A.) 10 μM **Tz** for 48 h; (B.) 20 μM **Tz** for 24 h; (C.) 20 μM **Tz** for 48 h; (D.) 50 μM **Tz** for 2 h; (E.) decrease of MFI of GFP relative to the untreated GFP-expressing HEK293 cells. All experiments were performed in duplicate. Error bars represent ± s.d.

We carried out a series of transfections of GFP-expressing HEK239 cells. In one experiment, the cells were cotransfected with **sgRNA 2** and commercially available mRNA that encodes the Cas9 gene for 72 h. In another experiment, the cells were analogously cotransfected with the same mRNA and **sgRNA 2** that was cloaked with **TCO-Im** for 2 h. After each transfection, the medium was replaced with fresh DMEM and the cells were allowed to grow for an additional 48 h. GFP expression of each subset of cells was analyzed by flow cytometry. The cells treated with **sgRNA 2** had significantly lower expression of GFP relative to the untreated cells (Figure S8A). As illustrated in Figure 4E, the mean fluorescence intensity (MFI) of GFP decreased by 59% relative to that of the untreated cells. Meanwhile, the cells transfected with cloaked **sgRNA 2** had a minimally perturbed level of GFP expression, suggesting that the cloaked **sgRNA 2** had attenuated activity (Figure S8B). We carried out optimization of uncloaking of the latter subset of cells by treating them with different concentrations of **Tz** (10–50 μM) for variable amounts of time (2–48 h). It was determined that **Tz** is minimally toxic to cells in this concentration range (Figure S7). **Tz** is soluble in aqueous media and cell permeable. Therefore, it was added directly to the cell growth media. After that, the media was replaced with fresh DMEM and the cells were allowed to grow for additional 48 h. As illustrated in Figure 4A-D, the level of GFP expression decreased relative to that of the untreated cells, suggesting that the sgRNA was uncloaked by **Tz** inside the cells. The flow cytometry experiments indicated that the best uncloaking was achieved upon treatment with 50 μM **Tz** for 2 h (Figure 4D). Under these uncloaking conditions, the MFI of GFP decreased by 49% relative to the untreated GFP-expressing HEK239 cells. In a control experiment, we confirmed that by itself, **Tz** does not have a strong impact on GFP expression. In this experiment, GFP-expressing HEK239 cells were treated with 10, 20, and 50 μM of **Tz** for 2 h and were allowed to grow for an additional 48 h. The flow cytometry histograms appeared to be similar to the untreated cells (Figure S9).

## Conclusion

In this report, we described an approach for temporal control of CRISPR-Cas9 gene editing using bond-breaking bio-orthogonal chemistry between TCO and Tz. We reported **TCO-Im** reagent capable of nonspecific acylation of sgRNA and attenuation of its activity. Cloaking was optimized in solution using a model 18-nt RNA oligonucleotide as well as sgRNA targeting the GFP gene. Cloaked sgRNA was shown to have low activity in solution as well as in GFP-expressing HEK293 cells. We also illustrated that cloaked sgRNA can be reactivated upon addition of a water-soluble and cell-permeable **Tz** reagent. The uncloaking proceeded in two steps and partially restored CRISPR-Cas9 activity in solution. Optimization of uncloaking of sgRNA with **Tz** was carried out in live HEK293 cells and analyzed by flow cytometry. The presented technology has a number advantages over other reported methods to control sgRNA’s activity, such as Staudinger reaction^21^ or isonitrile-Tz reaction.^18^ The bio-orthogonal chemistry between TCO and Tz is faster and more biocompatible.^23,28,29^ Both TCO and Tz are known to be nontoxic and well-tolerated under physiological conditions.^22,25,30^ We foresee that the described technology will find applications in the field of developmental biology where it will be utilized to silence genes of interest at different developmental stages.

## Supporting Information Available

Experimental details, MALDI-TOF and NMR spectra, sequences of sgRNAs, kinetic study, and cell viability study (PDF)
