# Supplementary material for: Reversible RNA Acylation Using Bio-Orthogonal Chemistry Enables Temporal Control of CRISPR-Cas9 Nuclease Activity
Source: ACS Chem Biol. 2024 Jul 25;19(8):1719–24. doi: 10.1021/acschembio.4c00117 (PMC11334111; doi:10.1021/acschembio.4c00117)
Supplement: Supplementary file 1 — cb4c00117_si_001.pdf [file cb4c00117_si_001.pdf]

SUPPORTING INFORMATION

Reversible RNA acylation using bio-orthogonal chemistry enables temporal control of CRISPR-Cas9 nuclease activity.

Bhoomika Pandit[1], Linglan Fang[2], Eric T. Kool[2] and Maksim Royzen[1]*

[1] Department of Chemistry, University at Albany, 1400 Washington Ave. Albany, NY 12222, USA
[2] Department of Chemistry, Stanford University, 450 Serra Mall, Stanford, CA 94305, USA
mroyzen@albany.edu

**Table of Contents**	**Figure and Scheme titles**	**Page**
Materials and Methods		S2-S4
Figure S1	Evaluation of kinetics of the reaction between (2*E*)-2-cyclooctene-1-carboxylate and **Tz**	S5
Table S1	Predicted MALDI peaks (negative mode) of the 18-nt model RNA.	S6
Figure S2	MALDI-TOF spectrum of the model RNA cloaked with **TCO-Im** for 1 h	S7
Figure S3	MALDI-TOF spectrum of the model RNA cloaked with **TCO-Im** for 2 h.	S7
Figure S4	MALDI-TOF spectrum of the model RNA cloaked with **TCO-Im** for 4 h.	S8
Figure S5	Uncloaking of model RNA with **Tz** for 1 h.	S9
Figure S6	Uncloaking of model RNA with **Tz** for 2 h.	S9
Figure S7	Viability of GFP-expressing HEK293 cells treated with variable concentrations of **Tz**.	S10
Figure S8	Flow cytometry analysis of CRISPR experiments targeting the GFP gene in HEK293 cells.	S10
Figure S9	Flow cytometry analysis of GFP-expressing HEK293 cells treated with different concentrations of **Tz**.	S11
Synthesis of **TCO-Im**		S12
NMR and ESI-MS Spectra		S13-S15

MATERIALS AND METHODS

All oligonucleotide solid phase syntheses were done on a 1.0 μmol scale using the Oligo-800 synthesizer (Azco Biotech, Oceanside, CA, USA). Solid phase syntheses were performed on control-pore glass (CPG-1000) purchased from Glen Research (Sterling, VA, USA). Other oligonucleotide solid phase synthesis reagents were obtained from ChemGenes Corporation (Wilmington, MA, USA). Phosphoramidites (TBDMS as the 2′-OH protecting group): rA was N-Bz protected, rC was N-Ac protected and rG was N-iBu protected. A, C, G, U phosphoramidites were dissolved in anhydrous acetonitrile (0.07 M) directly before use. m^1A, m^6A, s^2U and s^4U phosphoramidites were dissolved in anhydrous acetonitrile (0.15 M) directly before use. Coupling step was done using 5-ethylthio-1H-tetrazole solution (0.25 M) in acetonitrile for 12 min. 5′-detritylation step was done using 3% trichloroacetic acid in CH_2Cl_2. Oxidation step was done using I_2 (0.02 M) in THF/pyridine/H_2O solution.

For gel electrophoresis, 10X Tris/Borate/EDTA (TBE) buffer was purchased from Fisher Scientific Company L.L.C. (Waltham, MA, USA) and used with proper dilution. 30% Arcylamide/Bis-arcylamide solution (29:1) was purchased from Bio-Rad Laboratories, Inc. (Hercules, CA, USA). GeneRuler 1 kb Plus DNA Ladder (cat.# FERSM1331) was purchased from Fisher Scientific. Chromatographic purifications of synthetic materials were conducted using SiliaSphere™ spherical silica gel with an average particle and pore size of 5 μm and 60 Å, respectively (Silicycle Inc, QC, Canada). Thin layer chromatography (TLC) was performed on SiliaPlate™ silica gel TLC plates with 250 μm thickness (Silicycle Inc, QC, Canada). Flash chromatography was performed using Biotage Isolara One instrument (Biotage Sweden AB, Uppsala, Sweden). Preparative TLC was performed using SiliaPlate™ silica gel TLC plates with 1000 μm thickness. ^1H, ^{13}C and ^{31}P NMR spectroscopy was performed on a Bruker NMR at 500 MHz (^1H) and 126 MHz (^{13}C). All ^{13}C NMR spectra were proton decoupled. High resolution ESI-MS spectra of small molecules was acquired using Agilent Technologies 6530 Q-TOF instrument. MALDI-TOF experiments were performed at the Stanford University Mass Spectrometry facility, using Bruker Daltonik Microflex MALDI-TOF spectrometer equipped with an N_2 laser. RNA samples were plated on an MSP Anchorchip 96 target plate and mass spectra were recorded in linear negative mode. Matrix consisted of 0.3 M trihydroxyacetophenone in EtOH and 0.1 M aqueous ammonium citrate, which were mixed in a 2:1 ratio by volume. Kinetic experiments were carried out using CARY

RNA Sequences:

Model RNA
5′-AUCCUGCCGACUACGCCA-3′

sgRNA 1:
5′-GGGCGAGGAGCUGUUCACCGGUUUUAGAGCUAGAAAUAGCAAGUUAAAAUAAGGC
UAGUCCGUUAUCAACUUGAAAAAGUGGCACCGAGUCGGUGCUUUUU-3′

sgRNA 2:
5′-GGGCGAGGAGCUGUUCACCGGUUUUAGagcuagaaauagcaaGUUaAaAuAaggcuaGUccG
UUAucAAcuugaaaaagugGcaccgagucggugcuuuuu-3′

Capital letters indicate unmodified nucleotides, while small letters correspond to nucleotides containing 2′-OMe groups.

Kinetics Experiments

The kinetics of the reaction between (2*E*)-2-cyclooctene-1-carboxylate and **Tz** was monitored by UV-Vis spectroscopy at 520 nm. This experiment was conducted under pseudo 1st order conditions in a 1:1 solution of DMSO:PBS (pH 7.4) at 25 °C with concentrations of (2*E*)-2-cyclooctene-1-carboxylate (5 mM) and **Tz** (0.5 mM). Absorbance at 520 nm was measured every 10 seconds. The kinetic experiments were performed in triplicate. Data were analyzed in GraphPad Prism 10 Software. The observed pseudo 1st order rate constant was used to calculate the second order rate constant.

Cloaking Procedure:

In a sterile 200 µL PCR tube, combined 2 µL of **TCO-Im** (2 M in DMSO) and 1 µL of **sgRNA 1** or **sgRNA 2** (80 µM in H_2O). Diluted with DMSO (4 µL) and H_2O (3 µL) to a total volume of 10 µL. The reaction mixture was incubated at 37 ˚C for (1, 2, and 4 h). Cloaked **sgRNA 1** or **sgRNA 2** was precipitated by adding 10 µL of 3 M NaOAc followed by 90 µL of H_2O and 500 µL of 100% cooled EtOH. The mixture was incubated at -80 ˚C overnight and centrifuged (21000 RCF) for 1 h at 4 ˚C to obtain the RNA pellet. The pellet was washed with 75% EtOH, dried and resuspended in H_2O.

Un-Cloaking Procedure:

Uncloaking was carried out by combining 1 µL of **Tz** (1 mM in PBS) and 1 µL of cloaked **sgRNA 1** or **sgRNA 2** (5 µM in H_2O). The solution was placed in a thermoshaker at 37 ˚C for 2 h. After that, the uncloaked **sgRNA 1** or **sgRNA 2** were used directly for CRISPR experiments.

CRISPR-Cas9 in vitro DNA cleavage assay:

eGFP-N1 plasmid DNA (10 U/µL, 1 µL, NEB, R3510L) was diluted with water (16.87 µL) and NEB buffer 3.1 (10x, 2 µL). The plasmid was linearized directly prior to CRISPR with DraIII-HF (10 U/µL, 1 µL, NEB, R3510L). For the Cas9-mediated DNA cleavage assay, Cloaked and Uncloaked sgRNA (300 nM, 5 µL), Cas9 (1 µM, 0.3 µL, NEB, M0386S), Cas9 buffer (10x, 1 µL, NEB), linearized plasmid (20 nM, 1.5 µL) and H2O (2.2 µL) were mixed (final volume = 10 µL) and incubated for 16 h at 37 °C. CRISPR experiments were terminated by the addition of proteinase K (20 mg/mL, 0.5 µL) for 1 h at 37 °C. The reaction (10µL) was mixed with blue loading buffer (6x, 2 µL, NEB, B7703S) and loaded on a 1% agarose stained with ethidium bromide (1x TBE running buffer).

CRISPR-Cas9 experiments in HEK293 cells:

Cloaked sgRNA

CRISPR-Cas9 experiments, were carried out following the procedure reported by Yin, H. et al. [Nat. Chem. Biol. 2018, 14, 311-316]. The GFP-expressing HEK293 cells were purchased from GenTarget (cat# SC001) and cultured in DMEM, containing 10% FBS and 1X Penicillin/Streptomycin, at 37 °C, 5% CO_2, and 95% humidity. The cells were seeded at a concentration of 1 × 10^5 cells per well in 6-well plate 24 h prior to the experiment. The cells were transfected with Cas9 mRNA (500 ng, Thermo Fisher Scientific), GFP-targeting **sgRNA 2** (30 nM) or cloaked **sgRNA 2** (30 nM) using lipofectamine (1.5 µL) (Invitrogen™ LMRNA003) for 72 h in Opti-MEM reduced serum media. After 72 h, Opti-MEM was replaced with fresh DMEM and the cells were grown for additional 48 h. The cells were treated with trypsin for 5 min, collected by

centrifugation at 1000 RPM and suspended in PBS (1 mL). GFP expression was analyzed by flow cytometry. Data from 10^6 cells were acquired using a FACS Aria III cell sorter equipped with a 488 nm/blue coherent sapphire solid-state laser, 20 mW (BD Biosciences, San Jose, CA, USA). Data analyses were carried out using FlowJo software (Ashland, OR, USA), according to manufacturer's instructions. Parameters, such as MFI and the percentages of specific populations were quantified by histogram analysis.

Optimization of uncloaking in HEK cells

The GFP-expressing HEK293 cells were cultured in DMEM, containing 10% FBS and 1X Penicillin/Streptomycin, at 37 °C, 5% CO_2, and 95% humidity. The cells were seeded at a concentration of 1×10^5 cells per well in 6-well plate 24 h prior to the experiment. The cells were transfected with Cas9 mRNA (500 ng, Thermo Fisher Scientific), GFP-targeting cloaked **sgRNA 2** (30 nM) using lipofectamine (1.5 μL) (Invitrogen™ LMRNA003) for 72 h in Opti-MEM reduced serum media. **Tz** was added directly to the transfection media. Different concentrations of **Tz**, as well as durations of treatment were tested: 10 μM for 48 h, 20 μM for 24 h, 20 μM for 48 h, 50 μM for 2 h. Afterwards, Opti-MEM media was removed and fresh DMEM was added. The cells were grown for additional 48 h. The cells were treated with trypsin for 5 min, collected by centrifugation at 1000 RPM and suspended in PBS (1 mL). GFP expression was analyzed by flow cytometry. Data from 10^6 cells were acquired using a FACS Aria III cell sorter equipped with a 488 nm/blue coherent sapphire solid-state laser, 20 mW (BD Biosciences, San Jose, CA, USA). Data analyses were carried out using FlowJo software (Ashland, OR, USA), according to manufacturer's instructions. Parameters, such as MFI and the percentages of specific populations were quantified by histogram analysis.

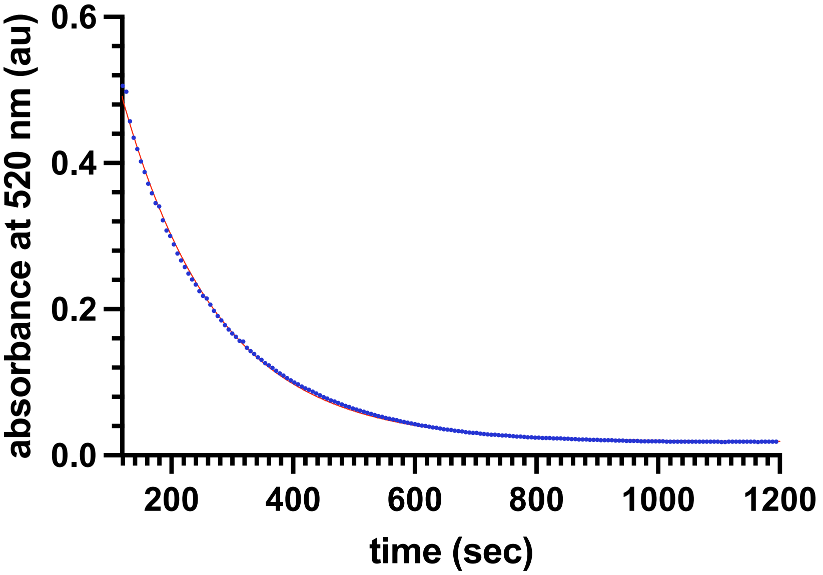

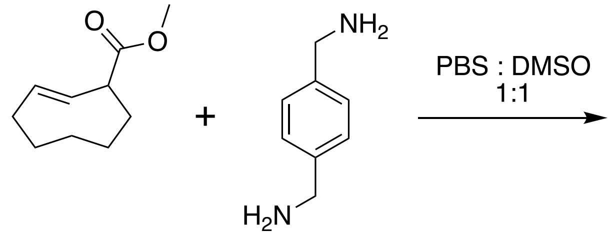

Figure S1. Evaluation of kinetics of the reaction between (2*E*)-2-cyclooctene-1-carboxylate and **Tz** performed in 1:1 solution of PBS (pH 7.4) and DMSO. Plot of absorbance at 520 nm, as function of time. The experiments were performed in duplicate. The shown data is representative of the duplicate experiments.

Number of TCO adducts			
0	+1	+2	+3
5647.7988 (100.0%)<br>5648.8022 (90.9%)<br>5646.7955 (54.7%)<br>5649.8056 (54.6%)<br>5649.8031 (25.3%)<br>5648.7959 (24.0%)<br>5650.8089 (24.0%)<br>5650.8064 (21.9%)<br>5649.7992 (21.8%)<br>5648.7997 (13.8%)	5783.8877 (100.0%)<br>5784.8910 (95.7%)<br>5785.8944 (60.7%)<br>5782.8843 (51.9%)<br>5786.8977 (28.2%)<br>5785.8919 (25.5%)<br>5786.8953 (24.4%)<br>5784.8847 (24.0%)<br>5785.8880 (23.0%)<br>5786.8914 (14.6%)	5920.9798 (100.0%)<br>5919.9765 (99.4%)<br>5921.9832 (66.6%)<br>5918.9731 (49.2%)<br>5922.9865 (32.7%)<br>5922.9841 (25.7%)<br>5921.9807 (25.5%)<br>5921.9769 (24.0%)<br>5920.9735 (23.9%)<br>5922.9802 (16.0%)	6057.0686 (100.0%)<br>6056.0653 (94.8%)<br>6058.0720 (69.9%)<br>6055.0619 (44.7%)<br>6059.0754 (36.0%)<br>6059.0729 (25.9%)<br>6058.0695 (24.6%)<br>6058.0657 (24.0%)<br>6057.0623 (22.8%)<br>6059.0690 (16.8%)
**Number of TCO adducts**			
**+4**	**+5**	**+6**	**+7**
6193.1575 (100.0%)<br>6192.1541 (90.6%)<br>6194.1608 (73.1%)<br>6191.1507 (40.9%)<br>6195.1642 (39.5%)<br>6195.1617 (26.1%)<br>6194.1545 (24.0%)<br>6194.1583 (23.7%)<br>6193.1511 (21.8%)<br>6196.1651 (17.8%)	6329.2463 (100.0%)<br>6328.2429 (86.8%)<br>6330.2496 (76.4%)<br>6331.2530 (43.2%)<br>6327.2396 (37.5%)<br>6331.2505 (26.3%)<br>6330.2433 (24.0%)<br>6330.2472 (22.8%)<br>6329.2400 (20.8%)<br>6332.2563 (18.7%)	6465.3351 (100.0%)<br>6464.3317 (83.3%)<br>6466.3384 (79.6%)<br>6467.3418 (47.0%)<br>6463.3284 (34.5%)<br>6467.3393 (26.5%)<br>6466.3321 (24.0%)<br>6466.3360 (22.1%)<br>6468.3452 (21.4%)<br>6465.3288 (20.0%)	6601.4239 (100.0%)<br>6602.4273 (82.9%)<br>6600.4205 (80.1%)<br>6603.4306 (51.0%)<br>6599.4172 (31.9%)<br>6603.4281 (26.7%)<br>6604.4340 (24.3%)<br>6602.4209 (24.0%)<br>6604.4315 (21.9%)<br>6602.4248 (21.4%)

Table S1. Predicted MALDI-TOF peaks (negative mode) of the 18-nt model RNA. Predicted peak heights are shown in the parentheses.

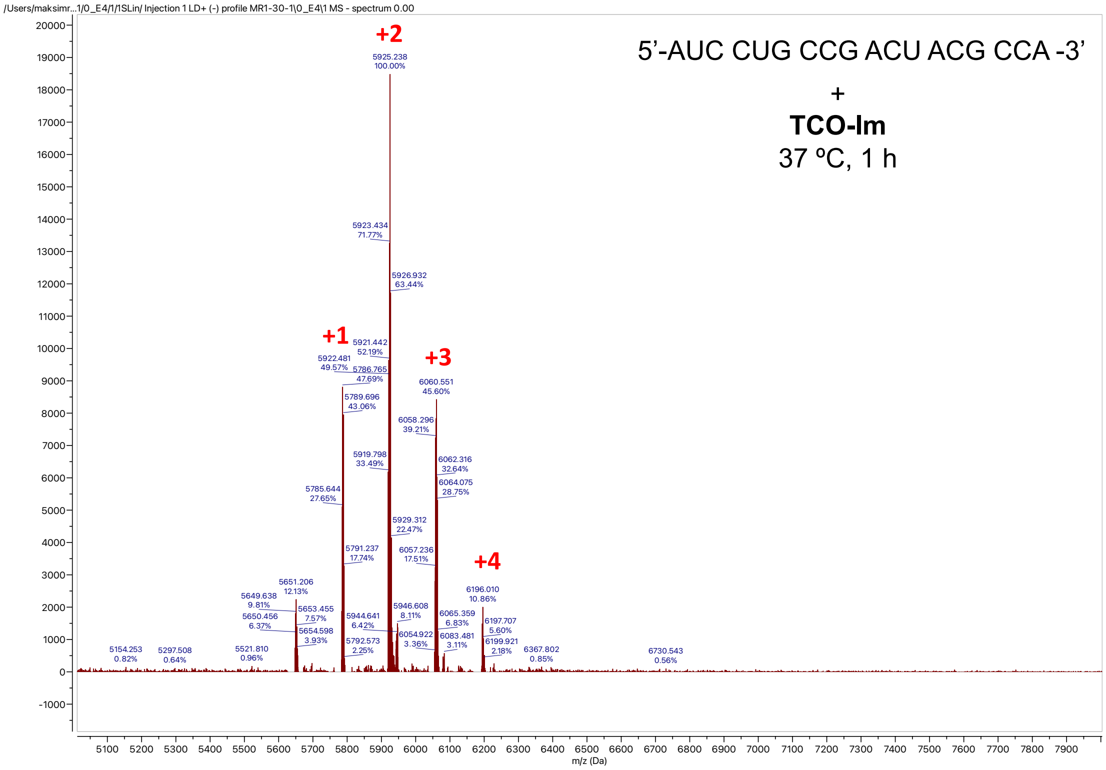

Figure S2. MALDI-TOF spectrum of the model RNA cloaked with **TCO-Im** for 1 h.

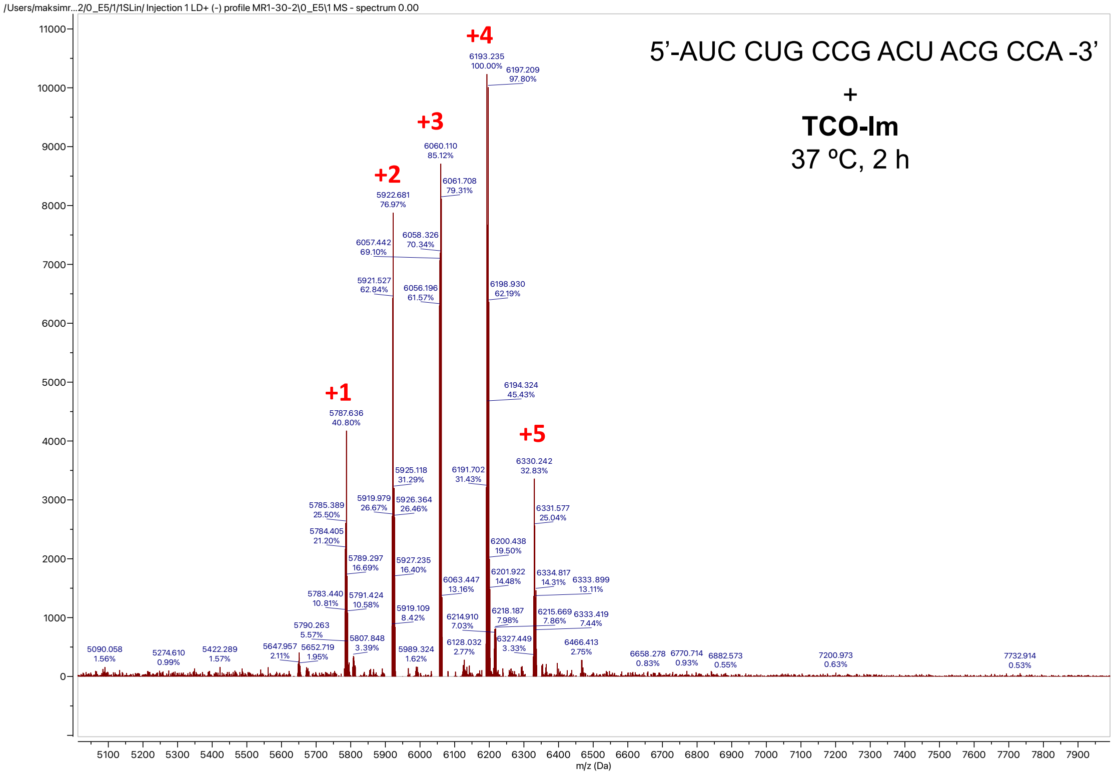

Figure S3. MALDI-TOF spectrum of the model RNA cloaked with **TCO-Im** for 2 h.

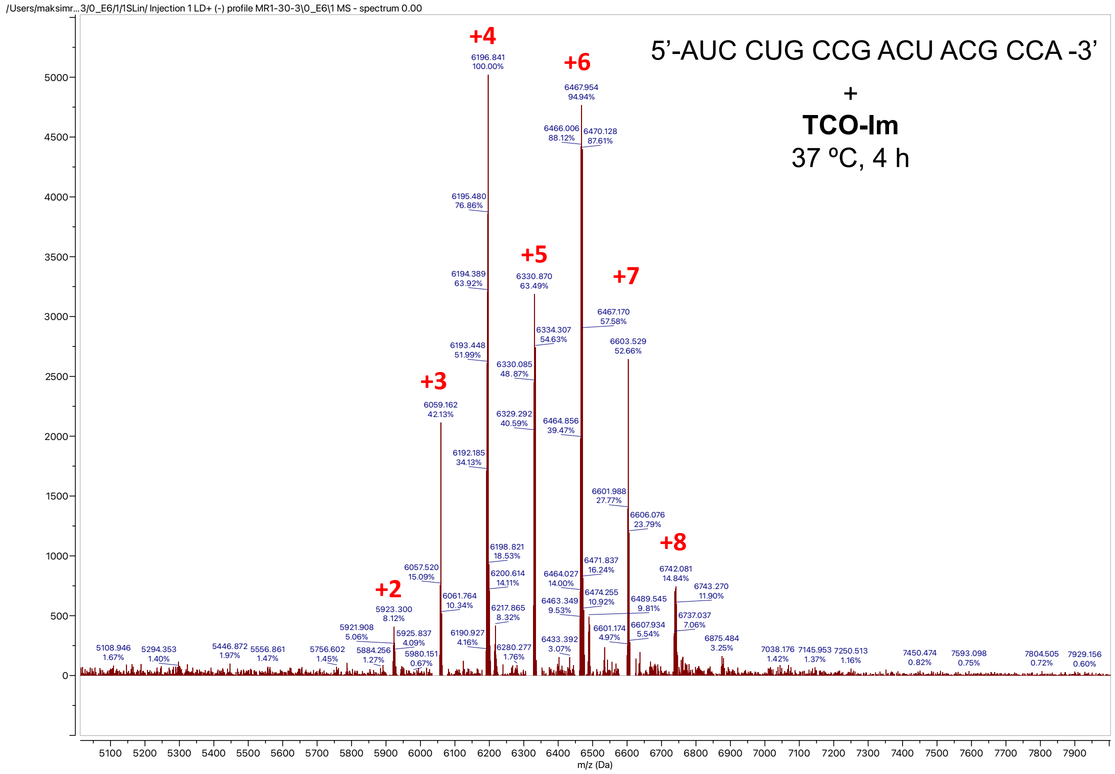

Figure S4. MALDI-TOF spectrum of the model RNA cloaked with **TCO-Im** for 4 h.

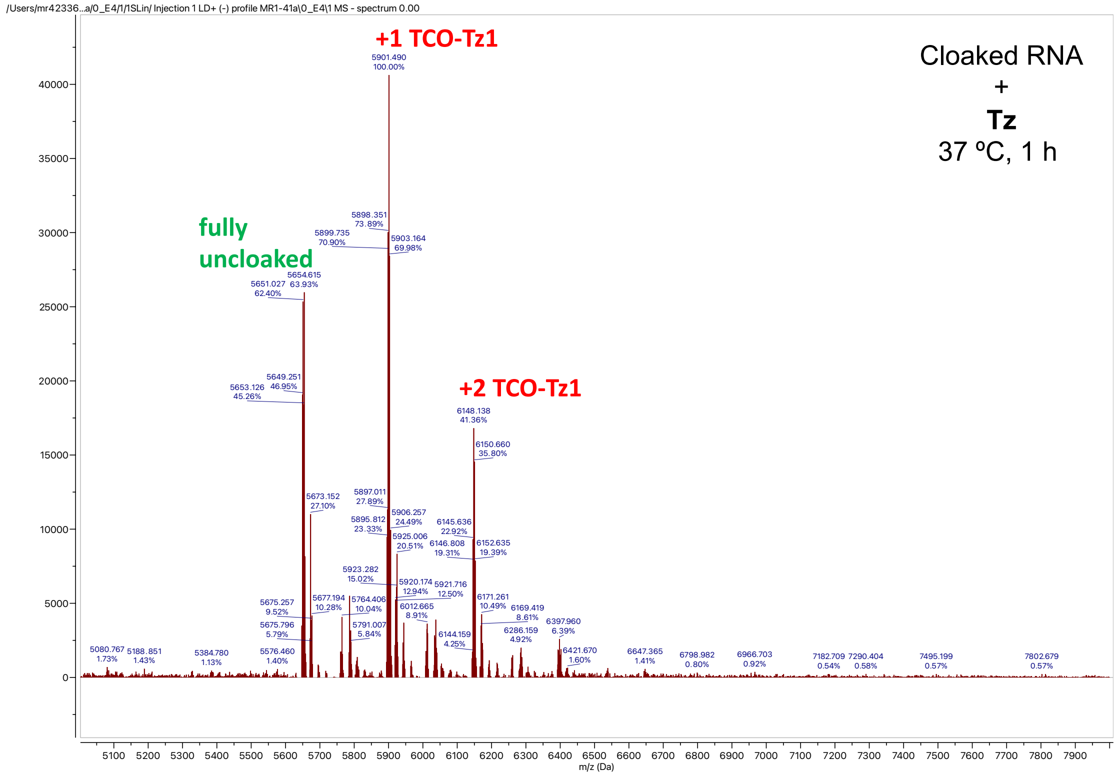

Figure S5. Uncloaking of model RNA with **Tz** for 1 h.

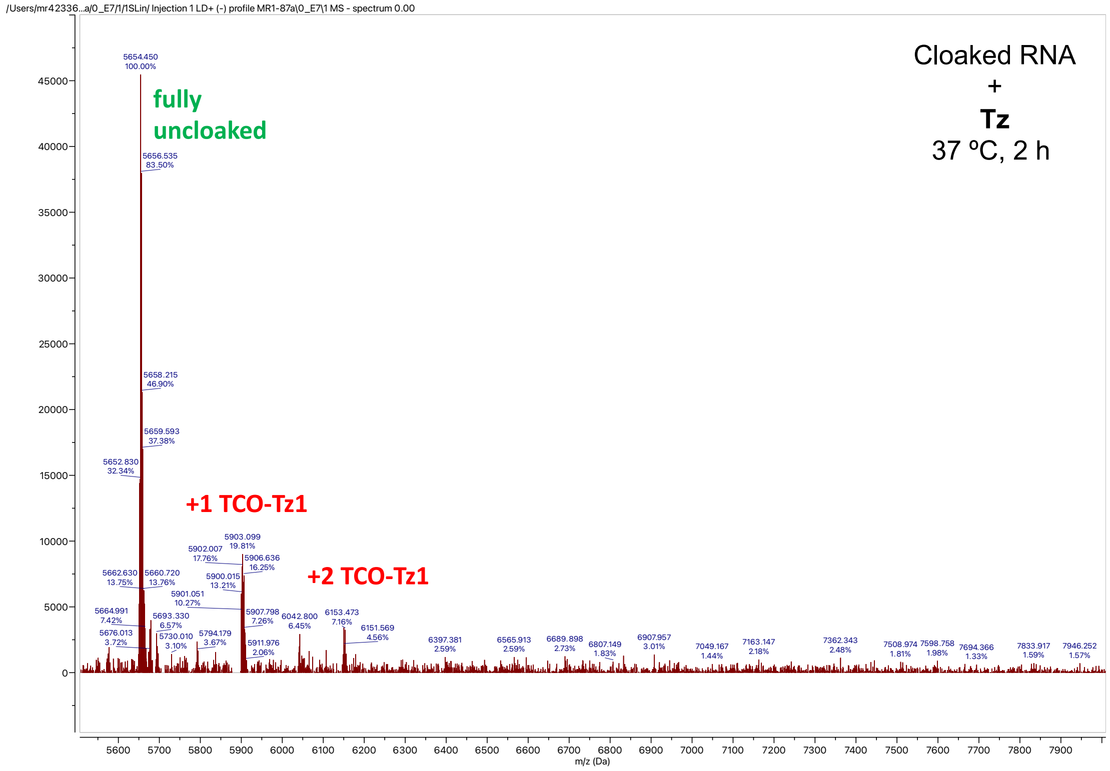

Figure S6. Uncloaking of model RNA with **Tz** for 2 h.

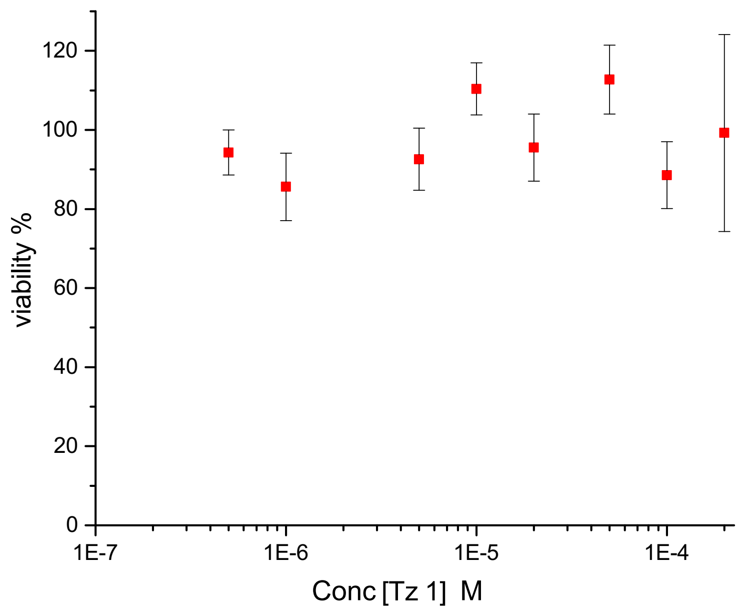

Figure S7. Viability of GFP-expressing HEK293 cells treated with variable concentrations of **Tz**. All experiments were performed in six replicates. Error bars represent ± s.d.

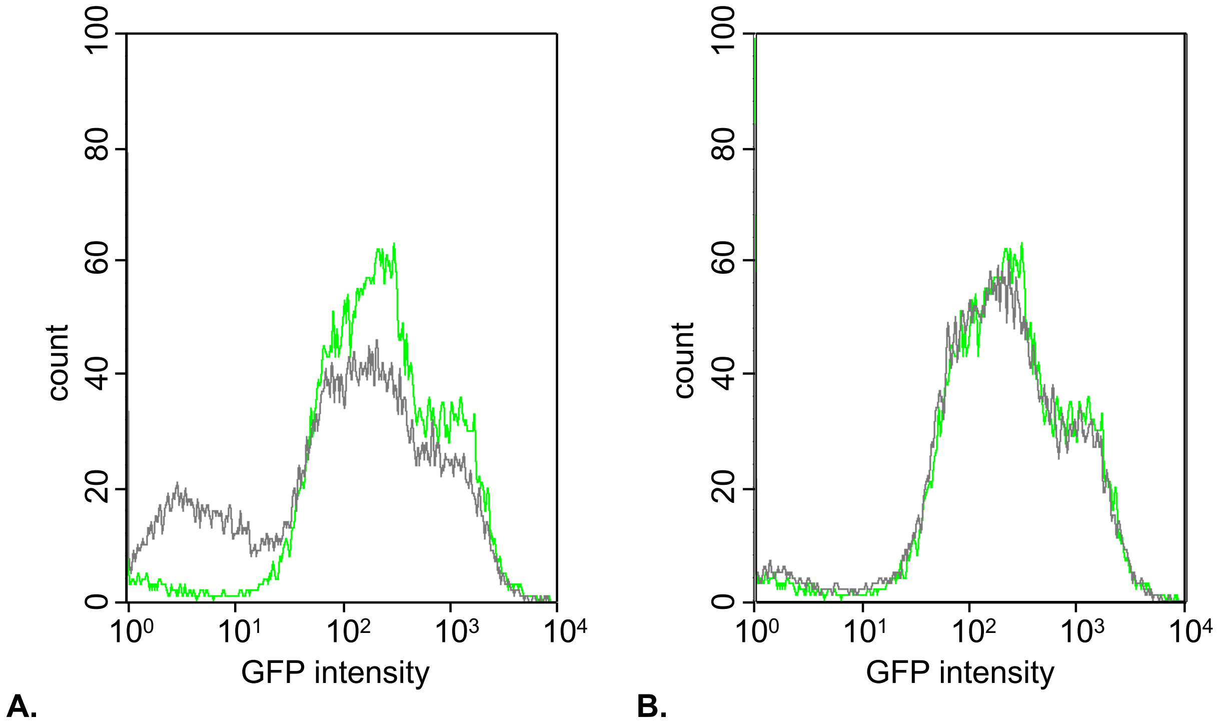

Figure S8. Flow cytometry analysis of CRISPR experiments targeting the GFP gene in GFP-expressing HEK293 cells. The experiments were performed in duplicate. The shown data is representative of the duplicate experiments. (**A**) Histograms of total GFP-expressing cells: untreated cells are shown in green, cells transfected with the **sgRNA2** are shown in grey. (**B**) Histograms of total GFP-expressing cells: untreated cells are shown in green, cells transfected with cloaked **sgRNA2** are shown in grey.

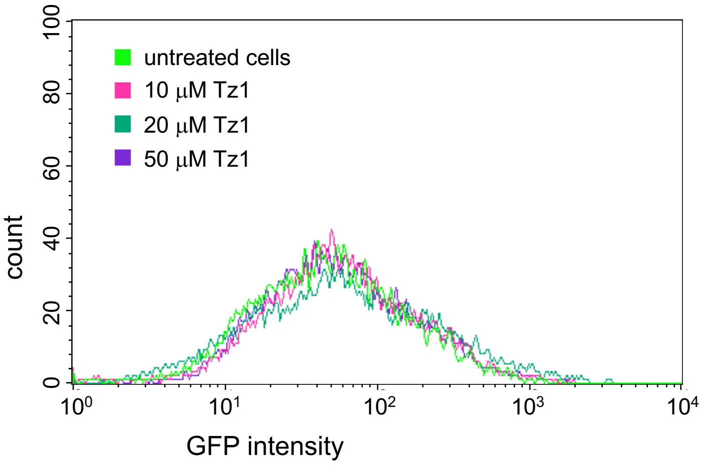

Figure S9. Flow cytometry analysis of GFP-expressing HEK293 cells treated with different concentrations of **Tz**. The experiments were performed in duplicate. The shown data is representative of the duplicate experiments.

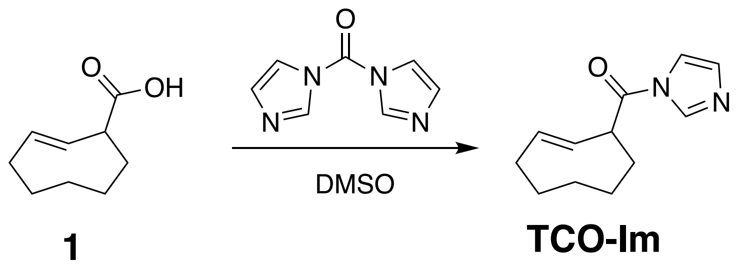

1

TCO-Im

Compound **1** was synthesized by following the reported procedure [*Org. Lett.* **2020**, *22*, 6041-6044]. Compound **1** was isolated as a mixture of axial and equatorial (eq) isomers in a 1:2.5 ratio. The obtained NMR spectra matched the reported ones. Dissolved **1** (100 mg, 0.65 mmol) in 162 μL of DMSO-d_6 to prepare a 4 M solution. Prepared a suspension of CDI (126 mg, 0.78 mmol) in 162 μL of DMSO-d_6. Dropwise added the suspension of CDI to the solution of **1** in DMSO-d_6. Agitated the reaction mixture for 5 min until the evolution of CO_2 was complete. The resulting 2 M DMSO solution of the crude product was used for RNA cloaking experiments without further purification. NMR spectra is a mixture of of axial and eq TCO isomers in a 1:2.5 ratio, plus one equivalent of imidazole:

^{1}H NMR (500 MHz, DMSO-d_6) δ 8.46 (s, 1H, axial), 8.37 (s, 1H, eq), 7.73 (s, 1H, axial) 7.66 (d, *J* = 4.4 Hz, 1H, eq and imidazole), 7.08 (s, 1H, axial), 7.03 (s, 1H, eq and imidazole), 5.90 (dd, *J* = 16.9, 5.8 Hz, 2H, axial and eq), 5.61 – 5.52 (m, 1H, axial), 5.40 – 5.26 (m, 2H, axial and eq), 4.31 (t, *J* = 5.3 Hz, 1H, eq), 4.11 (d, *J* = 4.8 Hz, 1H, axial), 2.40 – 2.23 (m, 3H, axial and eq), 2.15 (d, *J* = 13.7 Hz, 1H, axial), 2.00 – 1.31 (m, 11H, axial and eq), 1.28 – 1.03 (m, 2H, axial and eq), 0.97 – 0.72 (m, 3H, axial and eq).

^{13}C NMR (126 MHz, DMSO-d_6) δ 171.1, 170.2, 137.2, 137.1, 136.7, 135.1, 134.9, 130.7, 130.3, 130.0, 129.9, 121.7 116.7, 51.1, 47.3, 37.9, 35.7, 35.2, 35.1, 34.8, 28.2, 27.7, 26.1 26.0

HRMS (ESI) cal'd for $C_{12}H_{17}N_2O$ $[M+1]^+$ 205.1335; found 205.1327

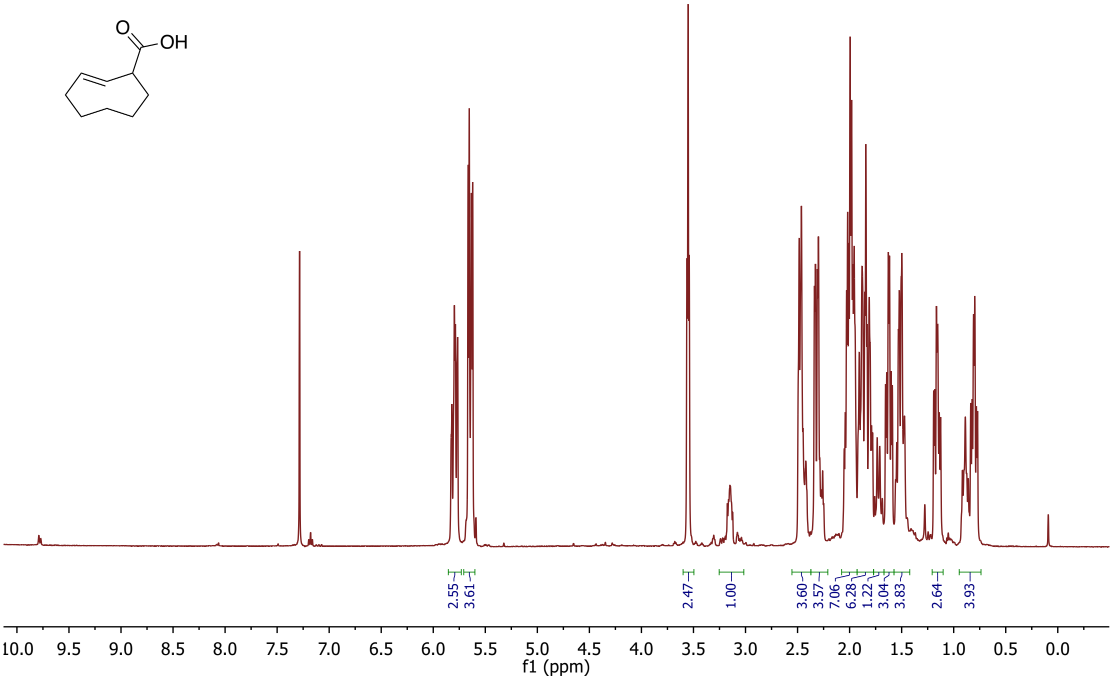
OH
O
2.55
3.61
2.47
1.00
3.60
3.57
7.06
6.28
1.22
3.04
3.83
2.64
3.93
10.0 9.5 9.0 8.5 8.0 7.5 7.0 6.5 6.0 5.5 5.0 4.5 4.0 3.5 3.0 2.5 2.0 1.5 1.0 0.5 0.0
f1 (ppm)

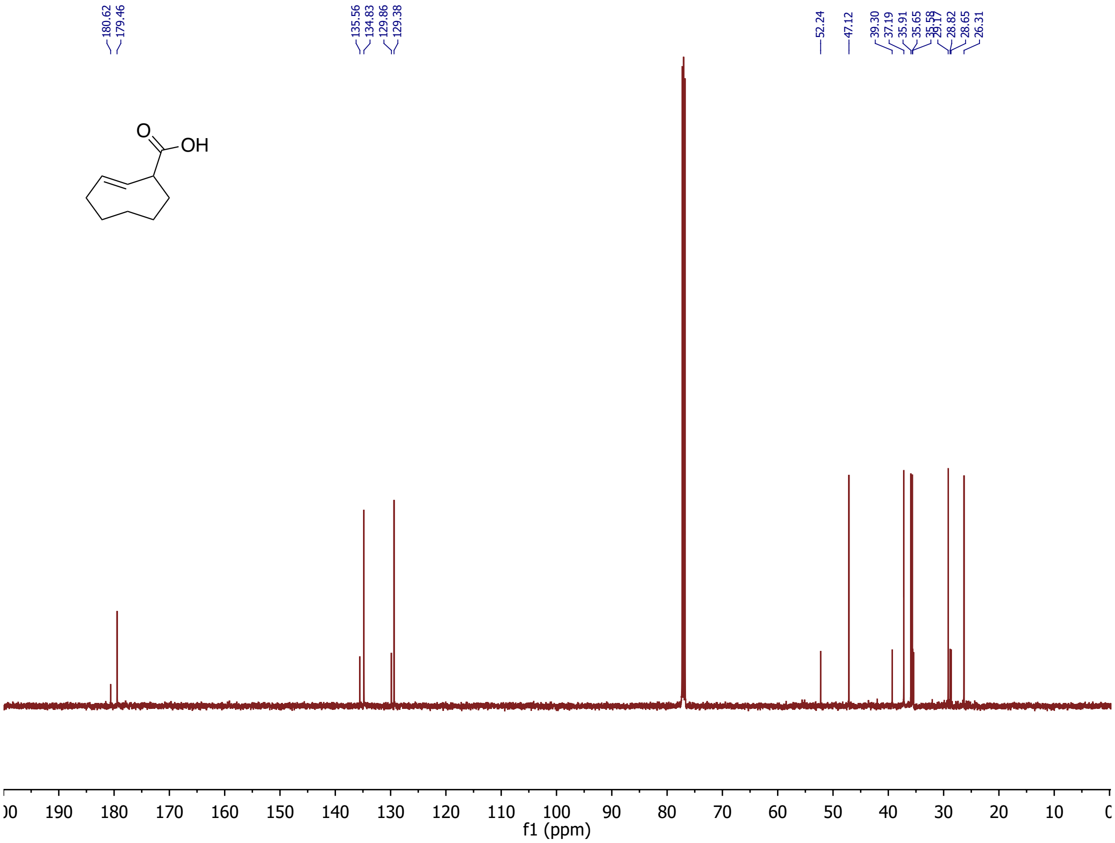
180.62
179.46
135.56
134.83
129.86
129.38
52.24
47.12
39.30
37.19
35.91
35.65
35.58
29.17
28.82
28.65
26.31
OH
O
190 180 170 160 150 140 130 120 110 100 90 80 70 60 50 40 30 20 10
f1 (ppm)

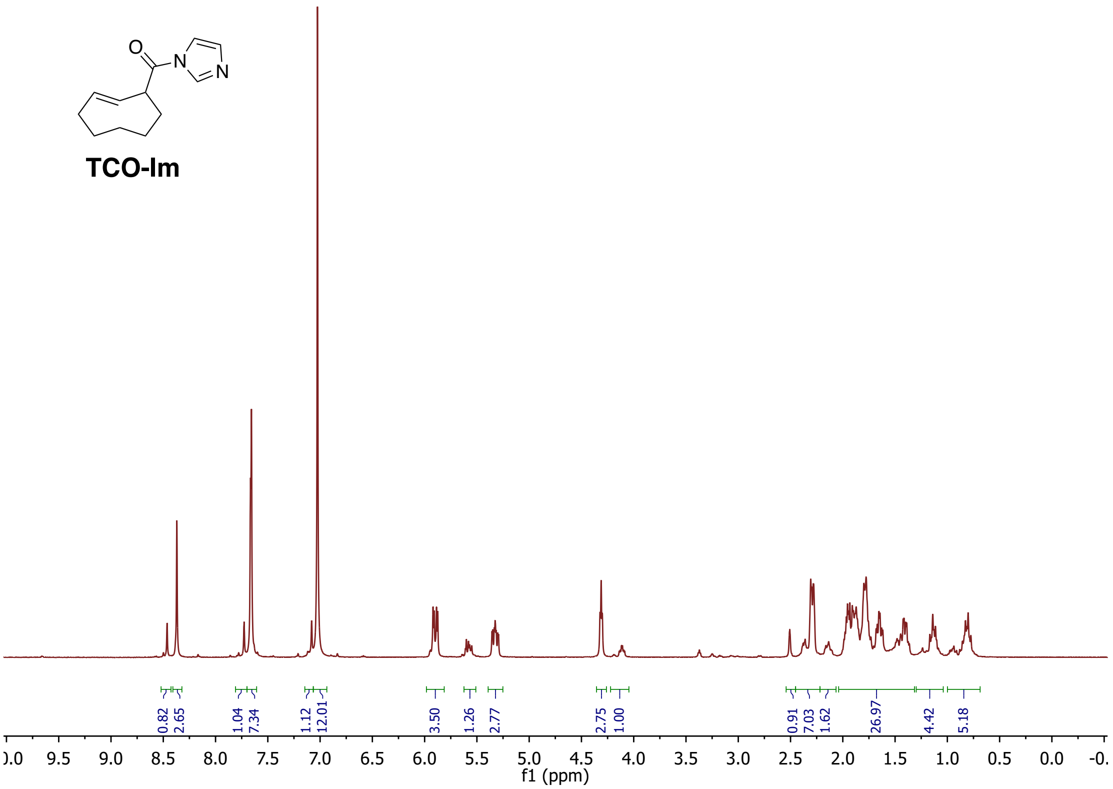

O
N
N
TCO-Im
0.82
2.65
1.04
7.34
1.12
12.01
3.50
1.26
2.77
2.75
1.00
0.91
7.03
1.62
26.97
4.42
5.18
f1 (ppm)

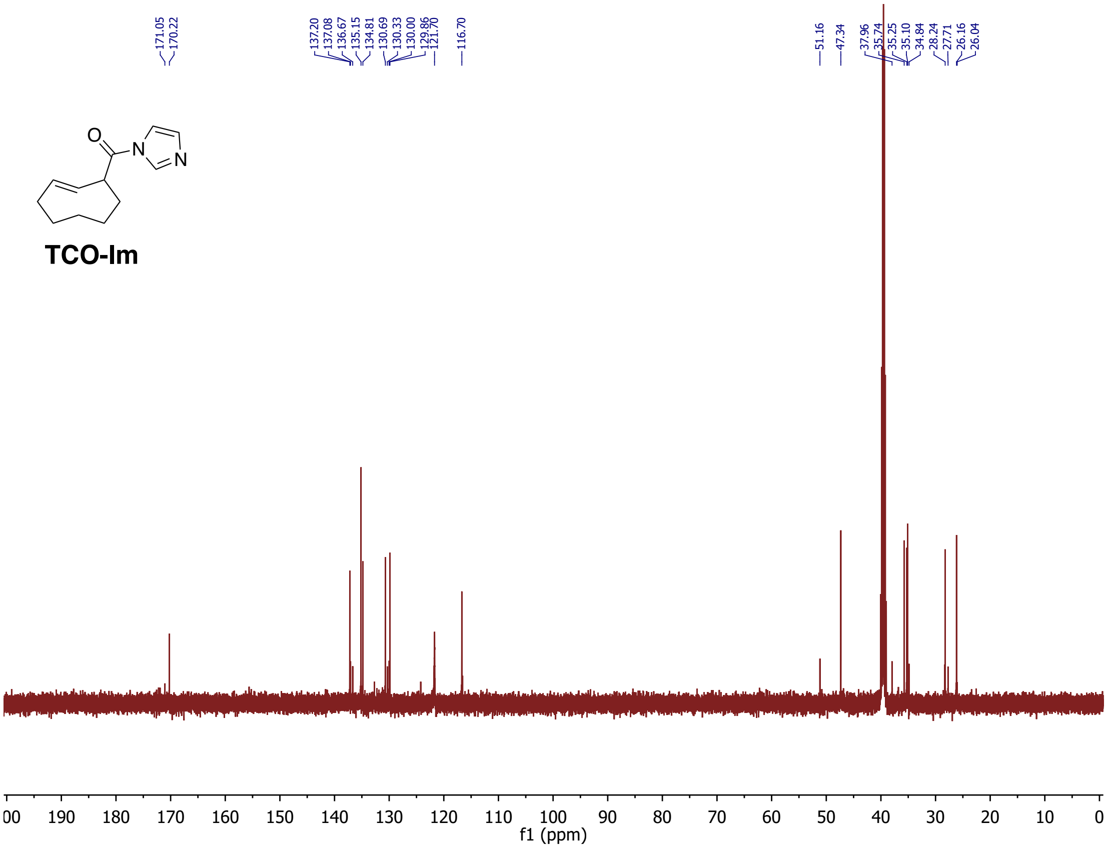

171.05
170.22
137.20
137.08
136.67
135.15
134.81
130.69
130.33
130.00
129.86
121.70
116.70
51.16
47.34
37.96
35.74
35.25
35.10
34.84
28.24
27.71
26.16
26.04
O
N
N
TCO-Im
f1 (ppm)

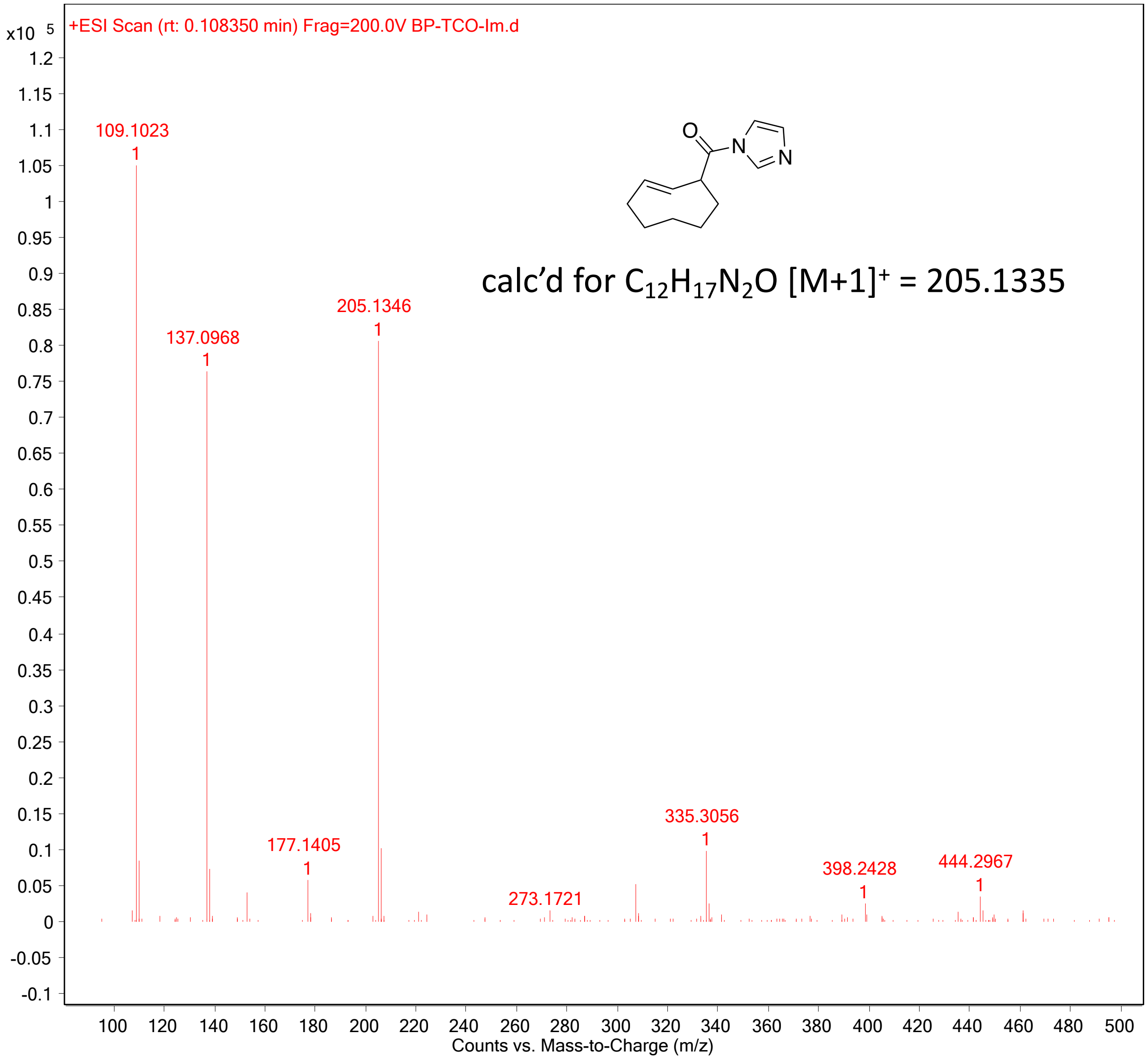

+ESI Scan (rt: 0.108350 min) Frag=200.0V BP-TCO-Im.d
x10 5
109.1023
137.0968
205.1346
177.1405
273.1721
335.3056
398.2428
444.2967
calc'd for $C_{12}H_{17}N_2O$ [M+1]$^+$ = 205.1335
Counts vs. Mass-to-Charge (m/z)
